# Supplementary material for: K-mer clustering algorithm using a MapReduce framework: application to the parallelization of the Inchworm module of Trinity
Source: BMC Bioinformatics. 2017 Nov 3;18:467. doi: 10.1186/s12859-017-1881-8 (PMC5670514; doi:10.1186/s12859-017-1881-8)
Supplement: Additional file 1: — Details of the MapReduce kmer clustering approach, and explicit algorithms for each step of the procedure. (PDF 73 kb) [file 12859_2017_1881_MOESM1_ESM.pdf]

K-mer clustering algorithm using MapReduce frame: application to the parallelization of Inchworm module of Trinity

Chang Sik Kim[1,†], Martyn D. Winn[1*], Vipin Sachdeva[2] and Kirk E. Jordan[2]

[1]The Hartree Centre, STFC Daresbury Laboratory, Warrington, WA4 4AD, UK

[2]Computational Science Center, IBM T.J. Watson Research, Cambridge, MA USA

[†]Cancer Research UK Manchester Institute, The University of Manchester, Manchester, M20 4BX, UK

[§]Silicon Therapeutics, 300 A Street, Boston MA, USA

[†,§]Present address

*Corresponding author: martyn.winn@stfc.ac.uk

Supplementary Methods

Algorithm 1: MR-step1 for extracting all unique k-mers and its abundant value. Note edge represents the pair of k-mers appearing consecutively in RNA-seq read; V_i represents the *i*th k-mer node; $E_{i,j}$ represents the pair of k-mers consecutively appearing on input reads; C_i represents the number of times k-mer_i found from all input reads; $CE_{i,j}$ represents the number of times $E_{i,j}$ found from all input reads.

**Input:**	1. MapReduce object *V* for storing k-mers<br>2. RNA-Seq input reads
**Algorithm:**	1. Map *V*: convert RNA-seq reads to k-mers<br>Input: Key = *null*, Value = *null*<br>i) Extract all k-mers from RNA-Seq reads<br>ii) Convert k-mer into 64 bit unsigned integer as V_i<br>Output: Key = V_i, Value = V_i<br><br>2. Collate *V*: k-mer as key<br><br>3. Reduce *V*: emit all unique k-mers<br>Input: Key = V_i, MultiValue = $V_1, V_2, \ldots, V_{Ci}$<br>C_i = the size of MultiValue<br>Output: Key = V_i, Value = C_i
**Output:**	1. MapReduce object *V*: Key = V_i, Value = C_i

Algorithm 2: MR-step2 for extracting all unique edges and its abundant value. See Algorithm 1 for the description of symbols in this algorithm.

Input:
1. MapReduce object E for storing edges
2. RNA-Seq input reads

Algorithm:
1. Map E: convert RNA-seq reads to edges
   Input: Key = *null*, Value = *null*
   i) Extract all pairs of k-mers as edges from RNA-Seq reads
   ii) Convert pairs of k-mers into (V_i, V_j)
   Output: Key = $E_{i,j}$= (V_i, V_j), Value = $E_{i,j}$

2. Collate E: edge as key

3. Reduce E: emit all unique edges
   Input: Key = $E_{i,j}$, MultiValue = $E_{i,j}$, $E_{i,j}$,..., $E_{i,j}$
   $CE_{i,j}$ = the size of MultiValue
   Output: Key = $E_{i,j}$ = (V_i, V_j, $CE_{i,j}$), Value = *null*

Output:
1. MapReduce object E: Key = $E_{i,j}$, Value = *null*

Algorithm 3: MR-step3 for edge filtering. See Algorithm1 for the description of symbols in this algorithm.

Input: 1. MapReduce object E: Key = $E_{i,j}$ = (V_i, V_j), Value = *null*

Algorithm: 1. Map E: convert edges to k-mer/edge pairs

Input: Key = $E_{i,j}$ = (V_i, V_j), Value = *null*

If *direction* = *right*:

Output: Key = V_i, Value = $E_{i,j}$

Else

Output: Key = V_j, Value = $E_{i,j}$

2. Collate V: k-mer node as key

3. Reduce E: emit the filtered edge assignment

Input: Key = V_i or V_j, MultiValue = $E_{i,j}$, $E_{i,j}$,…

CE_{max} = max(CE,CE,….)

If CE_i = CE_{max} for each E_i in Multivalue:

Output: Key = $E_{i,j}$ = (V_i, V_j), Value = *null*

Output: 1. MapReduce object E: Key = $E_{i,j}$, Value = *null*

Algorithm 4: MR-step4 for connected component labelling. See Algorithm 1 for the description of symbols in this algorithm.

Input:

1. MapReduce object E: Key = $E_{i,j}$ = (V_i, V_j), Value = *null*
2. MapReduce object V: Key = V_i, Value = Z_i = V_i
3. MapReduce object W = empty workspace

Algorithm:

1. Map W using E as input: convert edges to k-mer nodes
   Input: Key = $E_{i,j}$ = (V_i, V_j), Value = *null*
   Output: Key = V_i, Value = $E_{i,j}$ = (V_i, V_j)
   Output: Key = V_j, Value = $E_{i,j}$ = (V_i, V_j)
   Add V to W
   Collate W: V as key
   Reduce W: emit edges with zone ID of k-mer node
   Input: Key = V_i, Multivalue = *EEEE…Z* for each E in *MultiValue*
   Output: Key = $E_{i,j}$,, Value = Z_i

2. Collate W: Edge as key
   Reduce W: emit zone ID reassignment
   Input: Key = $E_{i,j}$,, Value = Z_iZ_j
   Z_{winner} = min(Z_i, Z_j); Z_{loser} = max(Z_i, Z_j)
   If Z_i and Z_j are different:
   Output: Key = Zloser, Value = Zwinner
   Exit: if W is empty

3. Map V: Invert k-mer node/zone ID pair
   Input: Key = V_i, Value = Z_i
   Output: Key = Z_i, Value = V_i

4. Map V using W as input: add zone ID assignments into W to V
   Output: Key = Z_i, Value = Z_{winner}
   Collate V: zone ID as key
   Reduce V: emit new zone ID assignment of each k-mer node
   Input: Key = Z_i, MultiValue = *VVVV…ZZZ…*
   Z_{new} = min(Z_i, Z_j, Z_k,…)
   For each V_i in MultiValue:
   Output: Key = V_i, Value = Z_{new}

Output:

1. MapReduce object V: Key = V_i, Value = Z_i

Algorithm 5: MR-step5 for construction of *inchworm* contigs. See Algorithm 1 for the description of symbols in this algorithm.

**Input:**	1. MapReduce object V_i: Key = V_i, Value = Z_i<br>2. MapReduce object K_i: Key = V_i, Value = C_i
**Algorithm:**	1. Add V to K<br>2. Collate K: k-mer node as key<br>3. Reduce K: emit zone ID with its k-mers and count values<br>Input: Key = V_i, MultiValue = Z_i, C_i<br>Create *hash_map* table: Key = V_i, Value = C_i<br>Sort *hash_map* table by C_i in descending order<br>Execute *inchworm* with *hash_map* table as input
**Output:**	1. *inchworm* contigs: assembled sequences in fasta format
